# Impact of embolization on stereotactic radiosurgery outcomes for intracranial arteriovenous malformations Spetzler-Martin grades III–V: a systematic review and meta-analysis

**DOI:** 10.3389/fsurg.2025.1563256

**Published:** 2025-04-03

**Authors:** Christopher Lauren, I Wayan Niryana, Tjokorda Gde Bagus Mahadewa

**Affiliations:** Neurosurgery Division, Department of Surgery, Faculty of Medicine, Udayana University, Ngoerah Hospital, Denpasar, Indonesia

**Keywords:** embolization, stereotactic radiosurgery, intracranial arteriovenous malformations, high-grade Spetzler Martin, obliteration rate

## Abstract

**Introduction:**

Intracranial arteriovenous malformations (AVMs) classified as Spetzler-Martin (SM) grades III-V present significant therapeutic challenges due to their complex angioarchitecture and high risk of morbidity. Stereotactic radiosurgery (SRS) is a minimally invasive modality for nidus obliteration, often combined with embolization to reduce nidus size and address high-risk vascular features. However, the impact of pre-SRS embolization on obliteration rates, post-SRS hemorrhage, and mortality remains controversial. This systematic review and meta-analysis aim to evaluate the effects of embolization on SRS outcomes in high-grade AVMs.

**Methods:**

Following PRISMA guidelines, a comprehensive search of PubMed, ScienceDirect, Cochrane, and Google Scholar was conducted. Studies comparing SRS alone versus SRS with embolization in SM grade III-V AVMs were included. Primary outcomes were obliteration rates, post-SRS hemorrhage, and mortality. Data extraction and quality assessment were performed using the Newcastle-Ottawa Scale, and pooled analysis was conducted using Review Manager (RevMan) software.

**Results:**

Out of 4,186 identified studies, five high-quality cohort studies met inclusion criteria. Pooled analysis showed that SRS alone resulted in higher obliteration rates than SRS with embolization (OR: 2.06, 95% CI: 0.92–4.65; *p*=0.08), though not statistically significant. Post-SRS hemorrhage rates were comparable (OR: 3.07, 95% CI: 0.72–13.08; *p* = 0.13), and mortality rates showed no significant difference (OR: 0.21, 95% CI: 0.01–4.62; *p* = 0.32).

**Discussion:**

Although embolization aids in nidus volume reduction, it may hinder radiosurgical efficacy by altering nidus architecture and introducing shielding effects. SRS alone demonstrated superior obliteration rates with fewer technical concerns. Individualized treatment planning remains essential, balancing embolization benefits against its potential drawbacks. Future studies should explore advancements in embolic agents and imaging techniques to optimize multimodal strategies for high-grade AVMs.

## 1 Introduction

Intracranial arteriovenous malformations (AVMs) are vascular anomalies defined by direct arterial-to-venous connections that bypass the capillary network. These abnormalities can cause considerable morbidity, including haemorrhage, convulsions, and neurological impairments. The mortality rates can arise up to 10%–15%, with morbidity rate of 50% and annual rupture risk of 2%–4% (1–3). The fundamental goal of AVM treatment is to accomplish total obliteration of the nidus, thus lowering the lifelong risk of rupture while maintaining neurological function. However, the complexity and heterogeneity of AVMs require a nuanced approach to treatment, especially for higher-grade lesions (1–3).

Among AVMs, Spetzler-Martin (SM) grades III–V reflect moderate to high-grade AVMs, defined by bigger diameters, prominent brain placements, and intricate venous drainage, which pose substantial obstacles to treatment and require thorough risk-benefit consideration (4). AVM management techniques include microsurgical resection, stereotactic radiosurgery (SRS), and endovascular embolization, which are usually used in conjunction for higher-grade tumours. Although microsurgery is the preferred treatment for lower-grade AVMs, it might be risky for eloquent or deep-seated SM grade III-V lesions. SRS has gained significance in these tough instances, presenting a minimally invasive approach to accomplish nidus obliteration over time, however with reduced success in bigger AVMs (5–7).

Embolization is frequently used as a neoadjuvant treatment to minimize nidus size or eliminate high-risk angioarchitecture features such intranidal aneurysms. Embolization for SM grades III–V improves the feasibility and safety of future radio surgical or surgical procedures. Despite these potential benefits, studies indicate that embolization alone seldom accomplishes complete obliteration, with rates largely depending on nidus complexity and the embolic agent utilized (8, 9).

The impact of pre-SRS embolization on obliteration rates remains uncertain, especially in moderate and high grade AVMs. Embolization may impair radio surgical efficacy by introducing radiopaque materials and increasing nidus recanalization, according to multiple investigations (7, 8).

Both embolization and SRS also can cause some dangerous complications. Embolization can cause consequences such as ischemia or embolic migration, resulting in neurological impairments in up to 40% of cases (10). SRS, on the other hand, increases the risk of radiation-induced alterations and latency-period haemorrhages, which might be worsened in partially embolized AVMs due to changed hemodynamic. The prognosis for SM grade III-V AVMs remain unsatisfactory when compared to lower-grade lesions. Radiosurgery alone has obliteration rates of less than 50% for AVMs larger than 3 cm in diameter (6, 8). Combined treatments integrating embolization have produced mixed results, with obliteration rates improving for smaller residual nidi but decreasing for bigger post-embolization AVMs (7). Embolization-induced physiologic alterations, including endothelial growth and nidus thrombosis, can disrupt the vascular response to SRS. Furthermore, technical issues such as difficulty targeting remaining nidus sites and changed dosage distributions contribute to heterogeneity in treatment outcomes (10).

This study aims to systematically analyse the impact of pre-SRS embolization on treatment outcomes for SM grade III–V AVMs, focusing on obliteration rates, post-SRS haemorrhage rate, and mortality. By synthesizing data from multiple studies, the aim is to provide evidence-based recommendations for optimizing multimodal treatment strategies for high-grade AVMs.

## 2 Methods

### 2.1 Study design and inclusion criteria

This systematic review and meta-analysis followed the Preferred Reporting Items for Systematic Reviews and Meta-Analyses (PRISMA) recommendations. Studies were eligible if they compared SRS alone to SRS combined with embolization for intracranial AVMs, reported at least one of the following outcomes: obliteration rates, post-SRS haemorrhage rates, or mortality, included SM grade III–V AVMs, and were cohort studies or randomized controlled trials with full-text availability in English. Exclusion criteria included studies with no clear categorization of treatment results by modality, studies focusing just on SM grades I–II AVMs, and those missing sufficient data for pooled analysis.

### 2.2 Literature search and selection

In December 2024, a complete literature search was undertaken using PubMed, ScienceDirect, Cochrane Central Register of Controlled Trials, and Google Scholar. Search tactics relied on Medical Subject Headings (MeSH) phrases and Boolean operators. Keywords used were “Arteriovenous malformation,” “Stereotactic radiosurgery,” “Embolization,” “Spetzler-Martin grade III–V,” “Obliteration rates,” “Post-SRS haemorrhage,” and “Mortality.” Mendeley software was used to identify and eliminate duplicate studies. To select suitable publications, two reviewers independently screened titles and abstracts before proceeding to full-text review. Disagreements were settled through conversation or by a third reviewer. The references of the listed publications were manually searched to find more relevant studies.

### 2.3 Quality assessment of included studies

The Newcastle-Ottawa Scale (NOS) was used to assess the quality of the included cohort studies. This tool analyses three domains, including selection (representativeness of cohorts and the determination of exposure), comparability (control for confounding factors, such as AVM grade and baseline characteristics), and outcome (adequate follow-up and reliability of outcome assessment). Each study was evaluated on a nine-point scale, with seven or higher signifying good quality. Two reviewers conducted independent assessments, and conflicts were resolved via consensus.

### 2.4 Data extraction, synthesis and statistical analysis

Data were gathered from all included research following a predetermined process to guarantee accuracy and uniformity. Key variables included patient demographics, including age, sex, and the distribution of Spetzler-Martin grades III–V AVMs, as well as study characteristics, including author details, publication year, and methodological design. Treatment-specific information was also recorded to enable reliable comparisons. Two investigators independently reviewed the extracted data and cross-checked it against the original articles to guarantee reliability. Any disagreements were discussed and, if required, a third reviewer was consulted. During extraction, clinical outcomes were given priority, with particular attention paid to primary outcomes such as obliteration rates, post-SRS bleeding rates, and mortality. When compiling the data, the authors of the original studies’ standardized definitions of outcomes were followed. This meticulous procedure guaranteed the integrity of the data that was collected, offering a solid basis for further synthesis and analysis.

The retrieved data were integrated using a thorough meta-analytic approach to determine pooled estimates and evaluate the relative effects of SRS alone vs. SRS plus embolization. Mortality, post-SRS haemorrhage rates, and obliteration rates were measured using effect measures such as odds ratios (ORs) with 95% CIs. A *p*-value threshold of less than 0.05 was used to determine statistical significance in the study, which was carried out using Review Manager (RevMan) version 5.4.1. Heterogeneity was measured using the *I*^2^ statistic to take into consideration possible variation amongst research. A random-effects model was used for more heterogeneity, and a fixed-effects model was used when *I*^2^ values were ≤50%, which indicates minimum heterogeneity.

## 3 Result

### 3.1 Study selection

This study complied with PRISMA guidelines to guarantee reproducibility in the selection of included studies with total of 4,186 records were first found. The remaining records were subjected to a thorough screening procedure based on title and abstract relevancy after duplicates were eliminated, and 596 studies were shortlisted for additional assessment. Based on their applicability to the study topic, which compared the results of SRS alone against SRS + E for Spetzler-Martin grade III–V cerebral arteriovenous malformations, 48 studies out of the 883 evaluated were requested for full-text retrieval.

**Figure 1:**
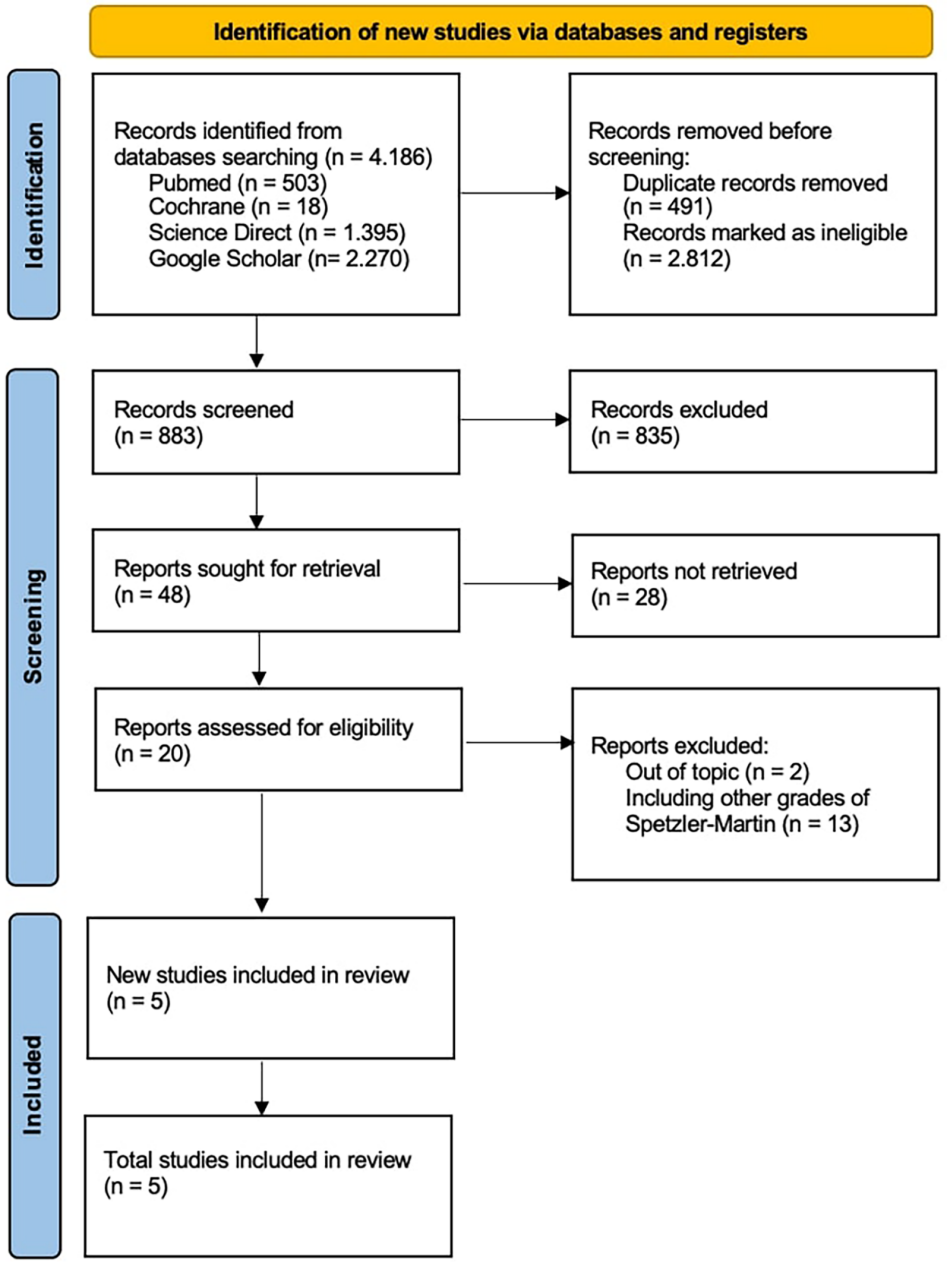
PRISMA algorithm.

According to the inclusion criteria, studies had to clearly stratify outcomes between SRS and SRS + E groups and provide death, post-SRS bleeding rates, and obliteration rates. Studies that only examined Spetzler-Martin grades I–II AVMs, had stratified outcomes, or had insufficient follow-up data were excluded. Five papers satisfied all inclusion criteria after a thorough eligibility evaluation, and they were added to the final meta-analysis (Figure 1).

### 3.2 Risk of bias analysis

The risk of bias analysis for the three included studies, assessed using NOS, reveals varying levels of methodological quality as shown in Table 1.

**Table 1:** Risk of bias analysis of included studies based on NOS.

Study	Selection (Max 4)	Comparability (Max 2)	Outcome (Max 3)	Total score (Max 9)	Risk of bias
Darsaut, 2011 (11)	4	2	2	7	Low
Hoh, 2000 (12)	3	1	2	6	Moderate
Marciscano, 2017 (4)	4	2	3	9	Low
Mizoi, 1998 (5)	3	1	2	6	Moderate
Yang, 2009 (10)	3	2	3	8	Low

Marciscano et al. (4) demonstrated the highest methodological quality among the studies, receiving a NOS score of 9/9. This study employed a representative cohort with clearly defined inclusion and exclusion criteria. Standardized imaging techniques such as MRI and angiography were utilized to validate diagnoses, and baseline characteristics were thoroughly described. The study also ensured strong comparability by controlling for variables such as AVM grade and treatment regimens. Furthermore, a follow-up period exceeding the minimum two-year requirement strengthened the reliability of its outcomes. Yang et al. (10) achieved a total NOS score of 8/9, indicating high methodological quality. Despite a slight deduction in the selection domain due to its single-institution focus, the study utilized a well-defined cohort. In contrast, Mizoi et al. (5), Darsaut et al. (11), and Hoh et al. (12) received a moderate NOS score, indicating a higher risk of bias. Eventhough they exhibit methodological constraints, their findings still provide insights into AVM obliteration patterns and long-term outcomes.

### 3.3 Baseline characteristics

A thorough framework for comparing the effects of SRS alone with SRS + E for intracranial AVMs is provided by the baseline characteristics of the three included studies in Table 2. These studies demonstrate the diversity of treatment modalities for high-grade AVMs by examining differences in population demographics, radiosurgical procedures, embolization techniques, and follow-up times.

**Table 2:** Baseline characteristics of included studies.

Author, year	Total participant	Age (years)	Country	Study design	SM grade	Radiosurgery	Embolic agent used	Follow-up duration	Obliteration confirmed via
Darsaut, 2011 (11)	44	Mean: 11.7 (overall)	United States	Retrospective cohort	IV–V	Charged-particle radiation, linear accelerator, CyberKnife, and Gamma Knife treatments, all performed as outpatient procedures	Thrombogenic coils, silk threads, polyvinyl alcohol particles, n-butyl cyanoacrylate glue, or Onyx liquid embolic.	6 months–4 years	MRI, Angiography
Hoh, 2000 (12)	24	Mean: 12.0 ± 5.8 (overall)	United States	Retrospective cohort	III–V	Stereotactic Bragg peak proton beam therapy (mean dose 15.9 ± 4.2 Gy, range 8.0–26.0 Gy, median 16.0 Gy)	n-butyl-cyanoacrylate	Mean: 38.7 months, (overall)	MRI
Marciscano, 2017 (4)	42	Median: 24.5	United States	Retrospective cohort	III–V	LINAC, Gamma NR Knife, CyberKnife, staged (median stages 2, median time between stages 3.5 years, median dose per stage 15.4 Gy, cummulative 33.5 Gy)	NR	Median: 9.5 years	SRS Angiography, SRS + E DSA, MRI
Mizoi, 1998 (5)	32	NR	Japan	Retrospective cohort	IV–V	Stereotactic subtraction angiography and MRI guidance (mean dose 19.2 Gy, range 12–25 Gy)	Ethanol and polyvinyl acetate solution	Mean: 45.7 months (24–58)	MRI, Angiography
Yang, 2009 (10)	46	Mean: 32.29	South Korea	Retrospective cohort	III–V	LINAC Fisher and Gamma Knife-based radiosurgery (mean dose 14.1 Gy, range 10–20 Gy)	n-Butyl cyanoacrylate (Histoacryl)	Mean: 78.1 months (34–166.4)	MRI, Angiography

SM, Spetzler-Martin; SRS, stereotactic radiosurgery; E, embolization; DSA, digital subtraction angiography; MRI: magnetic resonance imaging; LINAC, linear accelerator.

According to Marciscano et al. (4), 42 participants with a median age of 24.5 years received phased radiosurgical treatment. With a median dose of 15.4 Gy per stage and a cumulative dose of 33.5 Gy over a median interval of 3.5 years between stages, the radiosurgery protocols used in this study included LINAC, Gamma Knife, and CyberKnife. The median follow-up period was 9.5 years, and MRI, angiography, and SRS-specific digital subtraction angiography (DSA) were used to gain the results (4).

Darsaut et al. (11) described 44 patients, whose average age was 11.7 years, who were treated with Gamma Knife, CyberKnife, linear accelerator, and charged particle radiation. These were performed as outpatient treatments. Thromboembolic agents such as coils, silk threads, polyvinyl alcohol particles, n-butyl cyanoacrylate glue, or Onyx liquid embolic were used for pre-radiosurgical embolization. Obliteration results were validated by MRI and angiography during the follow-up period, which spanned six months to four years.

Hoh et al. (12) used stereotactic Bragg peak proton beam treatment on 24 subjects, whose mean age was 12.0 ± 5.8 years. With a median dose of 16.0 Gy and a range of 8.0–26.0 Gy, the mean dose was 15.9 ± 4.2 Gy. The average follow-up period was 38.7 months, and the embolic agent employed was n-butyl cyanoacrylate. Obliteration outcomes were confirmed by MRI.

Yang et al. (10), based in South Korea, examined 46 patients, whose mean age was 32.29 years, receiving radiosurgery using either a Gamma Knife or a linear accelerator (LINAC). The average radiosurgery dose was 14.1 Gy, with a range of 10–20 Gy. For pre-radiosurgical embolization, the study used n-butyl cyanoacrylate (Histoacryl) as the embolic agent. MRI and angiography were used to evaluate the results during the follow-up period, which lasted an average of 78.1 months (6.5 years) (10).

Mizoi et al. (5) focused on Spetzler-Martin grade IV and V AVMs and included 32 participants in Japan. The study used MRI guidance in conjunction with stereotactic subtraction angiography for radiosurgical planning, with a mean of 19.2 Gy and peripheral radiation doses ranging from 12 to 25 Gy. Ethanol and polyvinyl acetate solutions were used for embolization, mainly to reduce the size of the nidus prior to radiosurgery. The average follow-up period was 45.7 months, or over 4 years, and MRI and angiography were used to validate the results (5).

### 3.4 Obliteration rate

**Figure 2:**
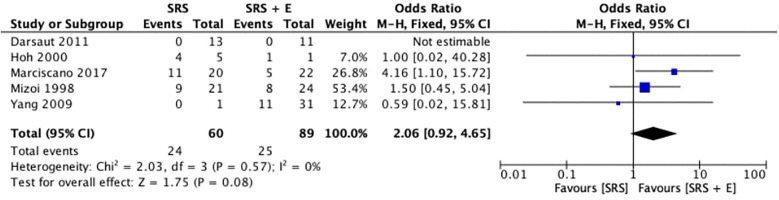
Pooled odd ratios of obliteration rates.

Five studies analyzed the obliteration rates of SRS alone in comparison to SRS plus embolization (SRS + E). With an overall OR of 2.06 (95% CI: 0.92–4.65; *p* = 0.08; *I*^2^ = 0%), the pooled odds ratio (OR) for obliteration favoured the SRS-alone group, as shown in Figure 2. This suggests that patients treated with SRS alone had a tendency toward higher obliteration rates. Nonetheless, the *p*-value for the total effect indicates that the difference did not achieve statistical significance, and the confidence interval crosses 1.0.

An *I*^2^ value of 0% and a Chi^2^ statistic of 2.03 (*p* = 0.57) demonstrate the low heterogeneity among the included studies, confirming the findings’ consistency. On an individual basis, Marciscano et al. (4) showed the biggest OR favouring SRS (4.16; 95% CI: 1.10–15.72). Moreover, Mizoi et al. (5) and Hoh et al. (12) reported a modest OR of 1.50 (95% CI: 0.45–5.04) and 1.00 (95% CI: 0.02–40.28), respectively, while Yang et al. (10) found no discernible obliteration benefit with an OR of 0.59 (95% CI: 0.02–15.81). On the other hand, Darsaut et al. (11) could not estimate the OR due to insufficient events in both groups. The results imply that SRS alone might be more successful than SRS + E in achieving obliteration, despite the absence of statistical significance.

### 3.5 Post-SRS haemorrhage

**Figure 3:**
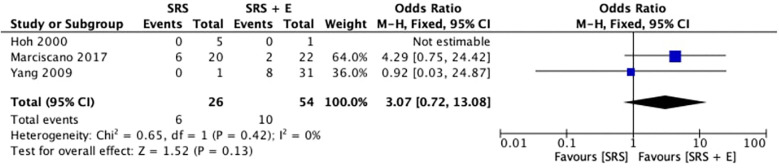
Pooled odd ratios of post-SRS haemorrhage.

Figure 3 shows the comparison of post-stereotactic radiosurgery (post-SRS) bleeding rates between SRS alone and SRS + E. The pooled OR was 3.07 (95% CI: 0.72–13.08; *p* = 0.13; *I*^2^ = 0%), favoring the SRS-alone group for post-SRS hemorrhage. However, the broad confidence interval and the overall *p*-value of 0.13 demonstrate that the difference was not statistically significant, despite the OR indicating a potential trend toward higher hemorrhage rates in the SRS + E group. An *I*^2^ value of 0% and a Chi^2^ statistic of 0.65 (*p* = 0.42) confirmed minimal heterogeneity among the studies, indicating consistency across the included research.

### 3.6 Mortality

**Figure 4:**
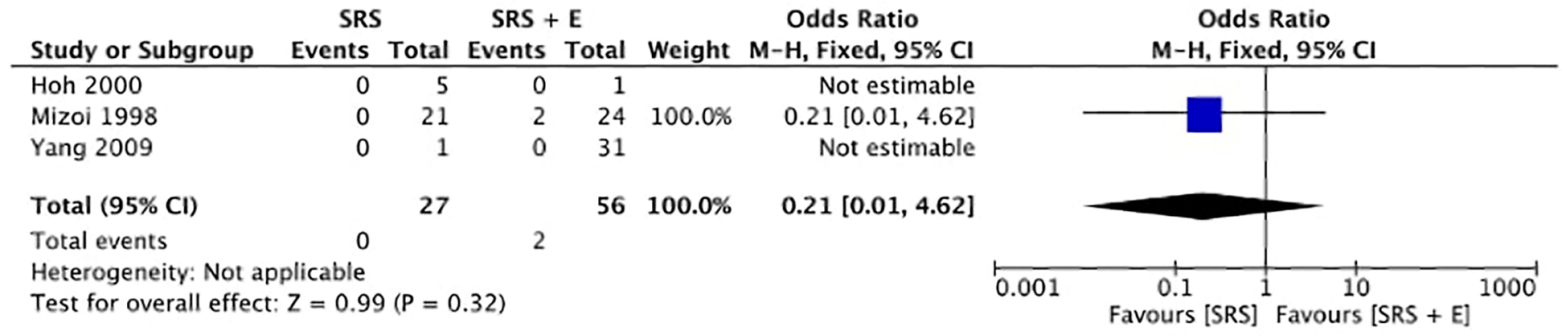
Pooled odd ratios of mortality.

Figure 4 illustrates the analysis of the mortality rates after SRS alone vs. SRS + E. The pooled OR was 0.21 (95% CI: 0.01–4.62), favoring the SRS + E group. However, the overall *p*-value (*p* = 0.32) and the confidence interval crossing 1.0 indicate that the difference in mortality rates between the two groups was not statistically significant. There is no recorded mortality in the SRS group and SRS + E group, so that Yang et al. (10) and Hoh et al. (12) were unable to generate a calculable OR due to the limited data available. This lack of events in some studies highlights limitations in drawing definitive conclusions.

## 4 Discussion

This study's results add to the increasing amount of data investigating how embolization affects the results of SRS for Spetzler-Martin grade III–V AVMs. According to Kano et al. (13), who reported lower obliteration rates in embolized AVMs due to factors like incomplete nidus targeting and shielding effects of embolic agents, our meta-analysis showed a trend of lower obliteration rates in AVMs treated with SRS + E compared to SRS alone (13). Furthermore, Andrade-Souza et al. (14) demonstrated a noteworthy reduction in obliteration rates for embolized AVMs (47%) in contrast to SRS alone (70%), which is in similar agreement with our findings. According to these results, embolization may reduce the size of nidus but may present difficulties that compromise the effectiveness of radiosurgical procedures.

Our analysis's pooled obliteration rates were greater for SRS alone, which is consistent with research by Marciscano et al. (4) that found that pre-SRS embolization was linked to lower obliteration rates because of issues like insufficient nidus targeting and the shielding effects of embolic materials. By altering the architecture of nidus, embolization may theoretically make radiosurgical results more difficult to achieve by obfuscating accurate radiosurgical targeting (4). Studies like those by Schwyzer et al. (15), which found that embolized AVMs had a much lower obliteration rate (33%) than non-embolized AVMs (60.9%), provide ample evidence of this phenomena. Furthermore, residual, non-obliterated AVM components are frequently left behind after embolization, which may change the hemodynamic environment and raise the risk of latency-period hemorrhages (15).

Despite these limitations, there are certain particular benefits to using SRS + E in combination in some situations. By decreasing the nidus volume, embolization can improve the radiosurgical treatment of big AVMs. But as Izawa et al. (8) point out, this advantage is frequently offset by the decreased radiosurgical efficacy, especially in high-grade AVMs. Additionally, Oermann et al. (7) found that while embolization before to SRS may lessen radiation-induced problems, it may not offer a substantial defense against hemorrhagic episodes.​

Our study showed no significant difference in post-SRS hemorrhage rates between the SRS and SRS + E groups, which is in line with Chen et al.'s (1) findings that post-SRS hemorrhagic events in embolized AVMs did not significantly increase. Although embolization does not increase the risk, it also does not offer any further defense against latency-period hemorrhages, according to the stability in hemorrhage rates (1). These results are consistent with Jiang et al.'s (16) observation that embolization decreased obliteration rates without changing the risk of bleeding following SRS.

In line with the meta-analysis by Xu et al. (3), which found no discernible difference in long-term survival between SRS alone and SRS + E cohorts in all grade Spetzler-Martin, our analysis showed no significant difference in mortality rates across treatment groups. The idea that embolization adds complexity but does not substantially change overall survival results when paired with radiosurgery is supported by this observation (3).

The function of embolization is still controversial. In studies like Chen et al. (1), Onyx embolization—which has gained popularity because of its lower recanalization rates and better handling—has shown results that are equivalent to those of non-Onyx agents. The wider literature, as example by Chang et al. (17), however, emphasizes the variation in results depending on embolization technique, nidus complexity, and treatment planning. According to these results, embolization is a useful technique for reducing nidus, but how it affects radiosurgical results depends largely on clinical and technical considerations (17).

The results emphasize that high-grade AVMs require customized treatment planning. The advantages of reducing nidus size must be balanced against the possible drawbacks of decreased radiosurgical efficacy by multidisciplinary teams. According to Lee et al. (18), careful planning and imaging are necessary to maximize results, especially in complex instances. The best way to combine radiosurgery and embolization in AVM therapy requires more prospective research. According to Kano et al. (13), advanced imaging methods including functional MRI and high-resolution angiography may enhance radiosurgical targeting and nidus delineation in partially embolized AVMs. Furthermore, the difficulties currently related to pre-SRS embolization may be lessened by the creation of innovative embolic agents with improved biointegration and decreased radiodensity.

Although there are no statistically significant differences in obliteration rates, post-SRS bleeding, and mortality between SRS alone and SRS + E, these results warrant careful interpretation. Statistical non-significance does not inherently imply the lack of a genuine effect; rather, it may be indicative of constraints related to sample size, variety in study design, and differences in treatment approaches. The broad confidence ranges identified in this meta-analysis indicate considerable uncertainty in effect estimates, highlighting the necessity for additional research. The intricacy of high-grade AVMs and individual anatomical differences may lead to treatment outcomes that are challenging to quantify using traditional statistical methods.

From a clinical standpoint, the observed outcomes—despite their failure to achieve statistical significance—indicate that SRS alone may provide higher obliteration rates, while SRS + E remains relevant in specific instances where embolization is required for angioarchitectural modification or hemorrhage risk reduction. Although embolization did not substantially affect the overall post-SRS hemorrhage rates, it may still be a useful tool in stabilizing high-risk AVMs by addressing intranidal aneurysms or flow-related abnormalities before radiosurgery. Additionally, certain studies included in this analysis have suggested that embolization may potentially facilitate safer radiosurgical dose planning by minimizing the size of nidus (4, 15). Nevertheless, the routine use of embolization as a pre-SRS intervention should be meticulously assessed on a case-by-case basis, as it also introduces factors such as inadequate nidus targeting and altered hemodynamics.

The suitability of pre-SRS embolization for AVMs is still significantly influenced by patient selection, as the heterogeneity of AVM characteristics has a significant impact on treatment outcomes. This decision-making process is significantly influenced by the magnitude and location of AVMs. SM grade IV–V AVMs were the primary focus of previous studies (5, 11). These AVMs are typically larger and more profoundly seated, which makes them more difficult to completely obliterate through SRS alone. In such instances, embolization may be beneficial in minimizing the size of nidus and reducing the risk of high-risk angioarchitecture features, such as intranidal aneurysms. Nevertheless, the efficacy of embolization may be contingent upon the accessibility of the nidus and the capacity to accomplish a significant volume reduction without compromising radiosurgical targeting, as demonstrated by studies such as Hoh et al. (12) and Yang et al. (10). Consequently, although SRS alone appears to have higher obliteration rates in this meta-analysis, pre-SRS embolization may still be advantageous for AVMs situated in high-risk or eloquent brain regions, where volume reduction is essential for the safe delivery of radiosurgical doses.

When selecting patients for combined therapy, it is imperative to carefully evaluate embolization-specific risks, despite the potential benefits. Ischemia and embolic migration continue to be substantial complications of endovascular treatment, with neurological deficits reported in as many as 40% of cases (10, 12). The embolic agents employed in the studies included in this review were diverse, spanning from n-butyl-cyanoacrylate, Onyx liquid embolic to polyvinyl acetate and ethanol, which may have contributed to the variations in post-SRS obliteration rates (5, 10–12). The radiosensitivity of the remaining nidus may be reduced as a result of vascular reactivity being altered by embolism-related ischemia. Furthermore, the risk of post-SRS hemorrhage is elevated by the unintended occlusion of draining vessels, which can result from embolic material migration. This variability underscores the necessity of a standardized embolization protocol to enhance the safety and efficacy of multimodal AVM management.

Another critical factor that affects treatment outcomes is the risk of latency hemorrhage as a result of embolization, particularly in various AVM subtypes. Although some studies suggest that embolization may stabilize AVMs with high-flow arteriovenous shunting, others suggest that partially embolized AVMs may have an increased rupture risk during the latency period before complete obliteration (4, 5). The embolization process can incite endothelial alterations, which can result in increased susceptibility to hemorrhagic events and vessel fragility. Additionally, the interpretation of long-term hemorrhage risks is complicate by the variations in follow-up duration among studies, which range from six months in Darsaut et al. (11) to more than nine years in Marciscano et al. (4).

These results highlight the necessity of individualized risk stratification when considering embolization before SRS, especially for large, high-grade AVMs with complex venous drainage patterns. Given the variability in AVM characteristics, embolization techniques, and radiosurgical protocols, personalized treatment planning is essential for optimizing outcomes. Rather than relying solely on statistical significance, clinicians should adopt a multidisciplinary approach that accounts for patient-specific factors, AVM morphology, and institutional expertise to ensure the safest and most effective treatment strategy.

Nowadays, Artificial intelligence (AI) is revolutionizing the management of AVMs by enhancing diagnostic accuracy, treatment planning, and outcome prediction. Machine learning algorithms, particularly deep learning models, have exhibited substantial potential in the segmentation and characterization of AVMs, thereby enhancing the accuracy of lesion detection and classification (19). Automated lesion segmentation can enhance workflow efficiency and reduce interobserver variability, particularly in complex AVMs where nidus boundaries are difficult to define, through the use of AI-powered tools. Additionally, AI-driven prognostic models that integrate radiomic analysis and clinical parameters have been created to forecast treatment outcomes, enabling clinicians to customize management strategies according to patient-specific risk factors. The integration of AI into AVM management is on the brink of enhancing patient outcomes, reducing procedural complications, and optimizing decision-making (19, 20).

Additionally, the diagnosis and treatment of AVMs are being significantly enhanced by the implementation of advanced imaging techniques. Innovations such as diffusion tensor imaging (DTI), high-resolution digital subtraction angiography (DSA), and quantitative magnetic resonance angiography (QMRA) have enabled a more thorough evaluation of the angioarchitecture of AVMs, thereby facilitating the selection of appropriate treatments. For instance, the spatial relationship between AVMs and critical neural pathways, such as the corticospinal tract, has been evaluated using AI-assisted fiber tracking based on DTI. This approach has been employed to facilitate more precise surgical planning and reduce the likelihood of postoperative deficits (19). On the other hand, despite these promising developments, challenges remain in fully implementing AI and advanced imaging in AVM management. Variability in imaging protocols, data quality, and computational models may affect the reproducibility and generalizability of AI-based predictions. Furthermore, retrospective analyses have demonstrated retrospectively that AI is highly accurate; however, prospective validation through large-scale, multicenter studies is required to verify its clinical utility. In order to guarantee equitable access to AI-assisted healthcare, it is also necessary to address ethical considerations, such as algorithm transparency and potential biases in training datasets (19, 20).

This study also has some various limitations. First, the number of included papers was low, indicating a lack of high-quality, comparative research on this topic. Only five high-quality studies were included in this meta-analysis, leading to low statistical power and limiting the ability to draw definitive conclusions. Furthermore, the included studies had a retrospective design, which could have introduced biases. Direct comparisons as well as meta-analytic synthesis are made more difficult by variations in radiosurgical protocols, embolization methods, and follow-up periods among research. To overcome these limitations, future research should focus on conducting larger-scale randomized controlled trials to provide more robust evidence on the impact of embolization in SRS for high-grade AVMs. Last but not least, our study only included data that had been published, which could introduce publication bias by leaving out unpublished or continuing research.

## 5 Conclusion

This meta-analysis suggest that SRS by alone has higher obliteration rates than SRS + E, demonstrating its effectiveness as a stand-alone treatment for individuals who are carefully chosen. Although embolization can help with high-risk angioarchitectural characteristics and nidus reduction, its effect on radiosurgical results is still a worry because of the altered nidus architecture and possible shielding effects of embolic materials. Additionally, there is no discernible difference between the two treatment regimens’ post-SRS bleeding or fatality rates, according to the research. This consistency in safety results emphasizes the value of customized treatment planning, in which the possible advantages of embolization must be carefully balanced against the difficulties it presents for radiosurgical targeting. When the nidus is amenable to direct radiosurgical targeting, we advise using SRS alone as the recommended treatment for high-grade AVMs. Careful preparation is essential when embolization is judged required in order to reduce its effect on the effectiveness of following radiosurgical procedures. Future breakthroughs in embolic materials and imaging technology could improve the efficacy and safety of combination treatments; more research is needed to assess these advancements in clinical practice.

## Data Availability

The original contributions presented in the study are included in the article/Supplementary Material, further inquiries can be directed to the corresponding author.
